# Surrogate Endpoints: CONSORT and SPIRIT Extensions

**DOI:** 10.1177/00220345241275479

**Published:** 2024-10-06

**Authors:** F. Schwendicke, N.S. Jakubovics

**Affiliations:** 1 Clinic for Conservative Dentistry and Periodontology, Ludwig-Maximilians-University Munich, Munich, Germany; 2 School of Dental Sciences, Faculty of Medical Sciences, Newcastle University, Framlington Place, Newcastle upon Tyne, UK

**Keywords:** clinical studies, clinical protocols, guidelines, meta-research, outcomes research, trials

The efficacy of health interventions is best determined through well-designed randomized controlled trials (RCTs). Such trials should be (1) properly planned, with trial protocols being used for appraising a study (e.g., by funders or ethics committees), conducting it (e.g., by trial teams), and evaluating it (e.g., by journals or agencies), and (2) reported systematically and comprehensively, increasing reporting quality and reducing risks of reporting bias. Using guidelines for trial protocols and trial reports, such as the SPIRIT and CONSORT statements (Moher et al. 2010; Chan et al. 2013) has been found to enhance trial quality and reporting. Both SPIRIT and CONSORT are generic and may not be applicable or sufficient for all trial types, leading to the development of several extensions, which can be found via https://www.equator-network.org.

Surrogate endpoints are commonly used in trials to enhance their efficiency by reducing follow-up duration, sample sizes, and costs. A surrogate endpoint is used as a substitute for a target outcome, that is, a “direct measure of how a patient feels, functions, or survives,” “of relevance to trial participants, patients, clinicians, trialists, or other stakeholders” (Manyara et al. 2022). Surrogate endpoints do not directly measure such clinical benefit or harm but are expected to “predict the treatment effects on clinical benefit or harm based on epidemiologic; therapeutic; pathophysiologic; or other scientific evidence” (Manyara et al. 2022). Many oral and dental diseases are chronic in nature and develop over months or years. The assessment of target outcomes such as tooth retention or loss emanating from dental interventions in RCTs would require years or even decades of follow-up, while surrogates can pick up any potential intervention effects early. Therefore, surrogate endpoints are commonly used in clinical trials in dentistry (Pannuti et al. 2020). For example:

- In periodontology, changes in probing pocket depths and clinical attachment level are used as surrogates for periodontal disease progression, arrest or reversal, and (more importantly to patients) tooth retention or loss.
- In operative dentistry, margin integrity or surface characteristics of restorations (e.g., as part of FDI criteria) are used as surrogates for restoration survival and, again more relevant to patients, tooth retention or loss.
- In endodontics, changes in the periapical index are assumed to be indicative for outcomes such as future pain or tooth retention or loss.

The use of surrogate endpoints, however, comes with significant uncertainty about the true intervention effects, oftentimes severely limited data on risks and harms due to smaller sample sizes and shorter follow-up periods, and overall challenges in interpreting findings appropriately and deducing clinical or policy implications. So far, specific guidance as to how to reflect on surrogate endpoints when planning or reporting trials is missing, which is why there have been calls for extending SPIRIT or CONSORT for trials using surrogate endpoints to (1) reflect on the rationale and validity of using surrogates, (2) highlight the impact of endpoint choice on the assessment of harm and the interpretation of a trial, and (3) increase transparency toward surrogate endpoints being used for readers of and participants in trials (Ciani et al. 2023).

The SPIRIT|CONSORT-Surrogate project aimed to develop extensions for SPIRIT and CONSORT to improve reporting of trial protocols and trials when using surrogate endpoints as primary outcomes. The recently published extensions (Manyara et al. 2024a, 2024b) are of relevance to dentistry, and the *Journal of Dental Research* (*JDR*) strongly encourages researchers to adopt them when planning and reporting relevant trials. *JDR* further urges reviewers and editors to consider the extension when assessing trials using surrogates as primary outcomes. Briefly, the extensions cover the following aspects:

Researchers should:

- highlight in the abstract and introduction that the primary endpoint was a surrogate (also if the primary outcome was a composite including a surrogate) and which target outcome it what assumed to reflect on;
- justify the use of a surrogate and its validity and evidence;
- discuss how the employed surrogate reflects on expected true intervention benefits, but, more so, risks and harms; and
- consider involving patients in choosing and defining outcomes, particularly for surrogates, and clearly communicate if trial participants were aware of the outcome being a surrogate.

Notably, the extensions provide a minimum set of reporting items that researchers should consider and do not indicate that trial designs should necessarily be changed. Instead, they may raise awareness toward the validity of the design early on and toward transparently laying out the potential consequences of endpoint choice. Overall, the use of SPIRIT and CONSORT and the available extensions are expected to improve the quality and reporting of trials and their interpretation and to decrease research waste (Glasziou et al. 2014). They may, indirectly, also help patients and the wider public to accurately understand trial findings (Sullivan et al. 2023). Researchers, reviewers, editors, and readers of the *JDR* are encouraged to appraise trials against these statements but should also be aware that bias may emanate from a wider range of sources than reporting and endpoint choice. We also encourage authors to consider the recently published OHStat guidelines (Best et al. 2024) for clinical and epidemiological studies in the oral health domain. Nevertheless, the principles of the SPIRIT|CONSORT-Surrogate project should be incorporated wherever studies use surrogate endpoints as primary outcomes.

## Author Contributions

F. Schwendicke, N.S. Jakubovics, contributed to conception, design, data acquisition, analysis, and interpretation, drafted and critically revised the manuscript. All authors gave final approval and agree to be accountable for all aspects of the work.
